# Cognitive behavioral therapy for patients with schizophrenia

**DOI:** 10.1192/j.eurpsy.2023.2177

**Published:** 2023-07-19

**Authors:** I. Binic, J. Petrovic, J. Antonijevic, D. Pancic, M. Zdravkovic, F. Petrovic

**Affiliations:** 1 Psychiatry, Psychiatry Clinic, Clinical Center Nis, Serbia; Medical Faculty, University of Nis, Serbia; 2 Psychiatry, Special Hospital for Psychiatric Diseases “Gornja Toponica”, Nis, Serbia; 3 Psychiatry, Medical Faculty, University of Nis; 4 Forensic Medicine, Medical Faculty, University of Nis, Nis, Serbia; 5 Radiology, Clinical Centre Nis, Medical Faculty, University of Nis, Serbia, Nis, Serbia

## Abstract

**Introduction:**

In treating schizophrenia, there is growing interest in introducing and renewing psychosocial therapies, including psychotherapy. In recent years, this has specifically entailed the adaption of particular cognitive behavioral therapy (CBT) approaches, which were previously only utilized for treating anxiety and mood disorders. The negative symptomatology of schizophrenia, which has proven to be especially difficult to treat, can be a challenge for CBT, particularly in terms of enhancing relationships with family and friends and work engagement.

**Objectives:**

The objective was to summarize the advantages of CBT treatment in schizophrenia briefly.

**Methods:**

Patients with schizophrenia frequently have comorbid problems, such as anxiety disorders (and disorders) and traumatic experiences, which can be effectively treated with CBT. In addition to pharmacological therapy, CBT is acknowledged as the gold standard in several countries for the treatment of schizophrenia. According to studies, combining CBT with medication can minimize psychotic symptoms.

**Results:**

Regarding treatment, Beck describes the use of typical CBT techniques: building trust and engagement; working collaboratively to understand the meaning of symptoms; understanding the patient’s interpretation of past and present events, particularly those that the patient believes are related to the development and persistence of his or her current problems; normalizing these experiences and educating the patient about the stress-vulnerability model, and socialization. Clarifying the emotional and behavioral repercussions of a delusion’s activation leads to an initial examination of the evidence-based on more peripheral interpretations. It is recommended to treat negative symptoms such as amotivation, anergia, anhedonia, and social disengagement with behavioral self-monitoring, activity scheduling, ratings of mastery and enjoyment, graded work assignments, and assertiveness training.

**Conclusions:**

In treatment settings where physicians are already utilizing high-quality psychoeducational materials to enhance adherence, an excellent foundation exists for introducing individual CBT for schizophrenia patients.

**Disclosure of Interest:**

None Declared

